# Fertigation: Nutrition, Stimulation and Bioprotection of the Root in High Performance

**DOI:** 10.3390/plants7040088

**Published:** 2018-10-23

**Authors:** Víctor García-Gaytán, Fanny Hernández-Mendoza, Ana Velia Coria-Téllez, Soledad García-Morales, Esteban Sánchez-Rodríguez, Luis Rojas-Abarca, Hadiseh Daneshvar

**Affiliations:** 1 Laboratorio de Análisis y Diagnóstico del Patrimonio (LADIPA), Colegio de Michoacán, A.C., Cerro de Nahuatzen 85, La Piedad 59699, Michoacán, Mexico; ana.coriatel@yahoo.com.mx (A.V.C.-T.); esanchez@colmich.edu.mx (E.S.-R.); qfblrojas@gmail.com (L.R.-A.); 2 Colegio de Postgraduados, Campus Montecillo, Carretera México-Texcoco km 36.5, Montecillo 56230, Texcoco, Estado de México, Mexico; hernandez.fanny@colpos.mx; 3 CONACYT—Centro de Investigación y Asistencia en Tecnología y Diseño del Estado de Jalisco, Camino Arenero 1227, El Bajío del Arenal, Zapopan 45019, Jalisco, Mexico; gmsoledad@gmail.com; 4 Collage of Agriculture and Natural Resource, University of Tehran, Karaj 3158777871, Alborz, Iran; h.daneshvar@ut.ac.ir

**Keywords:** fertigation, nutrition, roots, high production, stimulation, bioprotection, biotic and abiotic stress

## Abstract

Temperature changes, drought, frost, and the presence of pest and diseases place enormous stress on crops, which implies that the potential performance of these crops may be affected. One of the main goals for agronomists, horticulturists, growers, physiologists, soil scientists, geneticists, plant breeders, phytopathologists, and microbiologists is to increase the food production on the same cultivable area and to ensure that they are safe and of high quality. Understanding the biophysical changes in soil will help to manage the crop’s ability to cope with biotic and abiotic stress. Optimization is needed in the nutrition of crops, which involves the use of biostimulants to counter oxidative stress and the management of strain bioformulations (bacteria and fungi) that protect and stimulate roots for the acquisition of nutrients. The implementation of these strategies in fertigation programs improves crop yields. This article addresses the importance of the stimulation and the bioprotection of the root as a fundamental pillar in ensuring the high performance of a crop.

## 1. Introduction

Redox potential (Eh) and pH are the main drivers of soil–plant–microorganism systems. This was determined based on the hypothesis that the plants function physiologically inside an Eh–pH specific internal rank and alter the Eh–pH of the rhizosphere together with the microorganisms to guarantee cellular homeostasis [1]. This perspective is important in the crop production as it provides us strategies for achieving high yields. It also highlights that the plant nutrition, the temperature extremes, drought, frost, the presence of pest and diseases as well as the use of biostimulants in the fertigation programs for the high production management are necessary. For precision agriculture and the characterization of soil conditions, the Eh–pH electrical conductivity (EC) could be used as an indicator of soil quality [2]. Geneticists and plant breeders are focusing their studies on developing plants with roots that improve the crop productivity under drought conditions, which have specific root characteristics including a thin diameter, specific length, and length density [3]. Architectural traits under genetic control include basal-root gravitropism, adventitious root formation, and lateral branching. This trait is important for the acquisition of phosphorus from infertile soils. Genetic variation in rhizosphere modification through the efflux of protons, organic acids, and enzymes is important for the mobilization of nutrients, such as phosphorus and transition metals, and the avoidance of aluminum toxicity [4]. Plants have diverse organs with different functions and nutritional requirements [5]. Therefore, understanding the root development is of vital agronomic importance [6]. It has been suggested that the distribution of the roots in the soil can be improved through genetic manipulation of the root system architecture by optimizing the absorption of water and nutrients [7]. It is challenging to obtain the measurement of the root architecture in the crops and the evaluation of the changes. It has been previously demonstrated that the changes in roots result in higher efficiency and tolerance to stress [8].

## 2. Nutrient Solution in Fertigation

**Figure 1:**
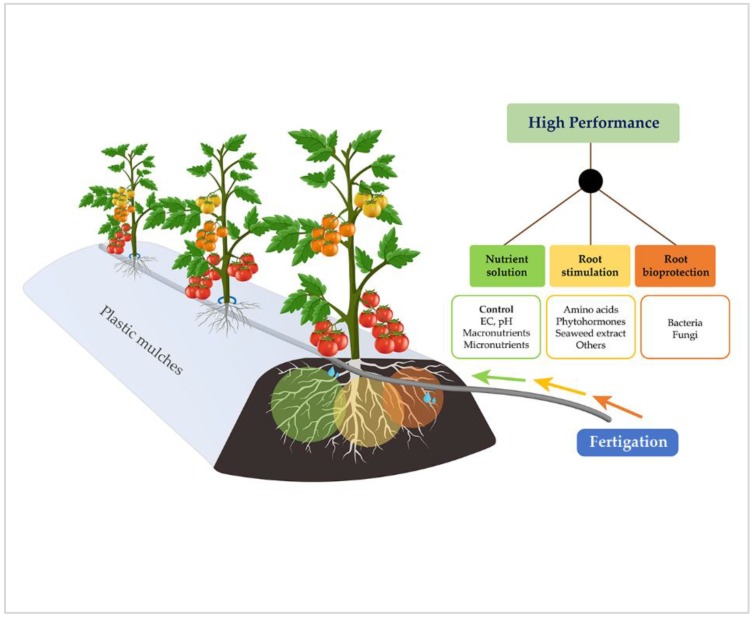
High crop production in the near future will be done through a good control of nutrient solution. In addition, we should consider stimulants (algae extract, protein hydrolysates, humic acids, phosphites, and phytohormones) and root bioprotection (fungi and bacteria) for greater nutrient acquisition and yield in improving fertigation programs.

The high yield and quality of crops is possible if nutrition is optimized. This includes the composition of the nutritive solution, water supply, nutrient solution temperature, concentration of dissolved oxygen, EC, and nutrient solution pH [9]. A high value of EC (3.5 and 4.5 dS m^−1^) increases the metabolites that are related to human diet (lycopene, ascorbic acid, phenols content, and antioxidant activity) [10], while an increase in EC reduces fruit cracking [11]. When the roots absorb excessive cations compared to anions, the roots offset this by excreting protons (H^+^), which generally leads to rhizosphere acidification. When they absorb more anions than cations, the roots excrete hydroxyl (OH^−^). Hydroxyl reacts with carbon dioxide to form bicarbonate (HCO_3_^−^), which leads to rhizosphere alkalization [12]. Fertigation is widely used in commercial and horticultural agriculture to produce high yield of high quality fruits and vegetables, with the combination of water and nutrients determined to be the key to success. Fertigation is not optional but it is indeed necessary for horticultural crops [13]. In fertigation, with the application of 56.4 cm of irrigation water and 3.4 kg ha^−1^ of urea daily, this results in higher performance of onion crops with less NO_3_-N leachate [14]. Better efficiency in the use of nutrients and water can be achieved through fertigation. A total of 25% of irrigation water can be saved and 33% more production can be obtained [15], while we can also maintain a low (but constant) nutrient level in soil solution, which is principally N [16,17]. An increase in fertigation frequency will allow us to reduce the concentration of immobile macronutrients, such as P, K, and micronutrients, and decrease environmental pollution [18]. Considering that nutrient leaching by fertigation is possible, the applied nutrients should not be submitted to excessive irrigation during application or in further irrigation [19]. Micronutrients, such as Fe, Mn, Zn, and Cu, may be applied through the irrigation system in chelated traces without causing any precipitation [20]. Through adequate management, it is possible to increase water use efficiency (WUE) by 25–40%. At the field level, these changes significantly increase the crop yield [21]. The nitrogenous fertilizers in the majority of crops is the most expensive, demanding and limiting ingredient for obtaining high yield, while the phosphate content varies at the soil micro-spatial level. Therefore, roots need to develop continuously to reach new soil sectors that are high in P [22]. P is the most immobile of the nutrients and K is relatively immobile, while Ca and Mg have an intermediate mobility [23]. The availability of N, P, K, and S limits low-input agriculture, while the phytoavailability of Fe, Zn, and Cu limits the crop production on alkaline and lime soil. Furthermore, P, Mg, Ca, and K deficiencies as well as Al and Mn proton toxicity limit the crop production in acid soil. Consequently, the development of genotypes that have a higher capacity for nutritional acquisition must increase yields in infertile soil [24]. Nutrient acquisition efficiency in soil is influenced by root proliferation, exuded carriers for nutrient activation, symbiotic associations, massive water flow, and ion spread over root surface [25,26]. Crop nutrition optimization in fertigation must involve a good balance of anion and cation, pH control, and EC of nutrient solution. The EC of this solution will be calculated based on the physico-chemical properties of soil and water quality. A challenge for agronomists in the world involves minimizing biotic or abiotic stress impact, which will be achieved through the usage of stimulants (vegetable or animal origin) and bioprotection (fungi and bacteria) in roots for a higher nutrient acquisition and high crop production as shown on Figure 1.

## 3. Biostimulants Usage in Crops

Another strategy for achieving high yields involves the usage of biostimulants, which include the vitamins and enzymes that are easily metabolized by microorganisms [27]. Biostimulants improve metabolic efficiency to induce an increase in yields and enhancement in crop quality; increase the plants’ tolerance and abiotic stress retrieval; and ease absorption, translocation and nutrient usage. Furthermore, this results in better quality attributes of products, including sugar content and color. Improving certain physicochemical properties of soil and building soil microorganism development contribute to the production of low-input crop [28,29]. Algae extract, protein hydrolyzates, humic and fulvic acids, and other compound mixtures have properties that are better than basic nutrition. They often improve growth and stress tolerance. Furthermore, all are vegetable biostimulants or bioefectors [30]. Biostimulant action is diverse, but it can include N metabolism activation, P release on soil, stimulation of soil microbial activity, and root stimulation [31].

### 3.1. Seaweed Extract

For the production of biostimulants, seaweed contains cytokines and auxins, which are essentially trace amounts of plant hormones [32,33,34,35]. The biologically active compounds that are transferred from seaweed biomass to the liquid phase during its fabrication include: polysaccharides, proteins, fatty acids, pigments, polyphenol, and minerals [36]. Mineral trace amounts present in seaweed extract act as enzymatic enhancers [34]. The benefits of seaweed application in the agricultural field are numerous: they stimulate germination, plant growth, root and stem elongation, water and nutrient absorption, frost resistance, biological control against phytopathogenic organisms, and contaminated soil remediation [37]. Micro-algae extract has a biostimulant effect in from the expression of characteristics of the roots and the genes related to nutrient acquisition [38]. In hydroponic systems, the micro-algae aggregation from the nutritive solution is possible as its use encourages the good development of the plants [39] and increases leaf photochemical efficiency, root length and dry weight, carbohydrates, K, Ca, and proline [40]. They have positive effects on attainment and profitability, while they also improve the content of proteins in corn grain under stress conditions [41,42]. In soy, the use of seaweed increased yield and a better absorption of N, P, K, Ca, and S was observed [43]. Furthermore, they have a reductive effect on abiotic stress, such as salinity, extreme temperatures, nutrient deficiency, and drought [44].

Seaweed and algae extract enhance the soil health by improving the capacity of moisture retention and developing microbe growth [45]. This improves the overall quality and certain characteristics, which includes: size, color, firmness, total soluble solids, ascorbic acid level, and minerals in tomato [46,47]. In corn, the algae extracts mainly stimulated the root growth, the nutrition uptake, the esterase activity, and sugar content [48]. New research proves that algae extract that is fortified with polysaccharides from the same algae resource can efficiently develop the growth of beans and tomato [49], while the combination of algae extract and 5-aminolevulinic acid increased flavonoid and antioxidants accumulation in *Asparagus aethiopicus* L. in saline [50].

### 3.2. Protein Hydrolyzates

Protein hydrolyzates are a group of important biostimulants that are based on a mixture of peptides and amino acids, which can be of vegetable or animal origin [51,52]. Protein hydrolyzates relieve the negative effects of abiotic stress from salinity, drought and heavy metals, and can stimulate the plant microbiome [53]. Under saline conditions, hydrolyzates increase yield in fresh, dry biomass and dry root weight in lettuce [54]. Hydrolyzate application in tomatoes improved the concentration of K and Mg in the leaf and net assimilation of CO_2_ [55]. Besides, they have a similar effect to auxins and gibberellin as they induce higher absorption of N and yield of corn, pea, and tomato [51]. With the application of 2.5 and 5.0 mL L^−1^, the percentage of germination, weight, and height in soy, tomato, and corn seedlings were improved [56].

### 3.3. Humic Acids

Humic acids are macromolecules that are a compound of humic substances. Commercial humic acids are extracted from the peat, which can be produced by fermentation and polymerization/condensation reactions. The humic substances are soluble in alkaline solutions, partially soluble in water and insoluble in acidic solutions [57,58,59,60,61,62,63]. The more labile and functionalized fraction of humic substances is responsible for root emission, while the more recalcitrant and less functionalized fraction of humic acids is related to root growth [64]. The most recalcitrant structures of the humic acids improve the preservation of organic matter in sandy soil [65]. Humic acids have a significant effect on soil fertility and are vital in the establishment of biotic and abiotic interactions in the plant’s rhizosphere [66]. The use of humic acids in the nutrient solution improves the root growth, with the absorption of nutrients including Ca, and increases the shelf life in *Gerbera jamesonii* L. [67]. When they are applied to soil, leaves, or before seeding, they significantly improve the grain yield in *Vigna radiata* L. [68], induce lateral root formation, and stimulate the micronutrient and macronutrient assimilation [69]. Furthermore, they induce tolerance to environmental stress [70] and successfully eliminates stress toxicity produced by Cd by modulating the status of water, photosynthetic apparatus, and antioxidant activity [71].

### 3.4. Phosphites (Phi)

Phi is a reduced form of phosphate (Pi), which is widely used as commercial fungicide and fertilizer or as biostimulants [72]. Phi is easily absorbed and transported through the xylem and the phloem to all parts of the plant and can be applied in many ways to the crops, such as fertigation, foliar spraying, log spraying, log injecting, surrow injecting, and soil flooding [73]. Potassium phosphite (KPhi) can be used as a protection strategy in crops against pathogens [74]. Phi improved and provided protection to cucumber plants against *Pythium ultimum*, and induced major yield and growth. The foregoing was related with higher induction of antioxidant enzymes (peroxidases, superoxide, dismutase and catalasas) [75]. Phi increase the positive regulation of various defense genes in jasmonate, salicylic acid and ethylene routs against *Phytophthora* [76]. Besides, it has an antibiotic effect on mycelial growth and the production of zoospores of the oomycete [77]. KPhi improves resistance through increasing the expression of defense molecules. The first events unleashed by KPhi are related to the enhancement of the cell wall. At the same time, the transcription factors StNPR1 and StWRKY act as coordinating amplifiers in the cascade in defense signaling [78]. Oxidative stress caused by UV–B radiation is reduced in plants that are pretreated with KPhi as this increases the defense mechanisms [79]. The application of KPhi before infection by pathogens efficiently activates the antioxidant system and eliminates the reactive oxygen species [80]. There was an increase in the flower number, foliar area, and P concentration in cucumbers [81]. Recently, innovative and promising research has been carried out on cotton plants. Transgenic cotton plants expressing the bacterial dehydrogenase phosphite gene (*ptxD*) are able to acquire the capacity to convert Phi into orthophosphate (Pi, the metabolizable form of phosphorus). Such plants allow for a selective fertilization scheme that is based on Phi as the sole source of P for the crop while offering an effective alternative for suppressing weed growth [82]. This technology has the potential to prevent the overuse of the limited Pi reserve and is environmentally sound. The Phi fertilizer use efficiency is close to 100% due its high solubility, reduced reactivity with soil components and non-utilization by most soil bacteria. These characteristics make Phi superior to conventional phosphate-based fertilization [73]. Using phosphite as a dual fertilizer and herbicide may mitigate the overuse of phosphorus fertilizers and reduce eutrophication and the development of herbicide resistance, which in turn will improve the sustainability of agriculture [83].

### 3.5. Plant Hormones

The use of plant hormones in crops is also a strategy to achieve high yields. For example, the application of indol-3-butyric acid (AIB) through fertigation at 0.5 L ha^−1^ doses increased pepper yields [84]. When auxins are applied in fertigation, the yield is significantly higher [85]. However, the response differed in growing melons (*Cucumis melo* L.) because the auxin application did not improve yield and the nutrients in crops [86]. Auxins play a crucial role in the regulation and development of different organs, including the root. They work as long- and short-distance signals and they coordinate cellular proliferation, cellular elongation, cellular differentiation, and endo-replication [87,88,89]. The response of the root architecture to nutrients can be modified through growth regulators, which suggests that nutritional control in root development can be mediated by changes in the synthesis and hormonal carriage [90]. It has been suggested that abscisic acid (ABA) accumulation modulates auxin carriage in the root, thus fostering root growth under water stress [91]. The lateral root formation and emergence in response to the phosphate availability is mediated by auxins [92]. Cytokinins regulate the auxin distribution in the root apical meristem [93] and function as a key regulator in stress tolerance [94].

## 4. Bioprotection in Plants

The microbial activity also stimulates plant growth through hormonal signaling, while plant growth-promoting rhizobacteria (PGPR) increase nutrient bioavailability in the soil and carbon cycle [22,95,96,97,98,99]. Most of the plants have an additional mechanism of nutrient acquisition (particularly N and P) [100,101,102]. These include beneficial microorganisms, such as bacteria, and fungi, which can be free-living, rhizospheric, or endosymbiotic [29]. The interaction between fungi and host plants is principally part of a nutrient acquisition strategy as the mycorrhizal fungi improve the nutrient absorption for plant growth. Therefore, the fungi is compensated by C compounds derived from photosynthesis [22,100]. At a defined pH range, the soil microorganisms may develop. For example, the bacteria population is higher in neutral soil and lower in acid soil [103]. Soil redox fluctuations, pH, and organic matter regulate N formation by *Azospirillum* spp. [104]. In phytopathogenic fungi, the *Aphanomyces cochlioides* and *Pythium* spp. impact was minor with an increase in pH [105]. When soil pH is increased to 7.0 and clay is added, it is possible to eradicate wilting due to *Fusarium oxysporum* [106]. On the other hand, the use of *Bacillus subtilis* can also reduce disease severeness caused by *Phytophthora* and increase the root length [107]. Other *Bacillus* genera are capable of reducing the root rotting caused by *F. oxysporum* by up to 70% [108]. Strain bioformulations of *Pseudomonas* and *Beauveria* effectively reduce pest and disease impact [109]. Therefore, its application in the form of special solution in the irrigation head should be considered in fertigation programs as a potential factor in obtaining high yields (Figure 1).

## 5. Conclusions and Perspective

Recent climate models predict that the impact and duration of drought and stress periods caused by heat are increasing in many regions of the world. This has a negative impact on the main crops, and consequently, the principal challenges at the international level involve improving the yield of crops under biotic and abiotic stresses. Furthermore, the genetic engineering of crop plants for enhanced salt tolerance will be a very important approach. At least 20% of all irrigated lands are salt-affected, with some estimates being as high as 50%. Attempts to improve the salt tolerance of crops through conventional breeding programs have met with very limited success due to the complexity of traits as salt tolerance is genetically and physiologically complex. Molecular genetics and functional genomics provide a new opportunity to combine molecular and physiological knowledge to improve the salinity tolerance of plants relevant to food production and environmental sustainability [110,111,112,113,114]. The current applied technology in food productions is not enough to ensure that the global population is fed. Promising research that enables a new green revolution will be related to root architecture, nutrient absorption, and nitrogen fixation [115]. Projections for agriculture in Mexico due to climate changes indicate that a reduction of 27% in national agro-food production will occur by the year of 2080 [116]. Therefore, it is necessary to develop efficient strategies to ensure food security [117]. Water scarcity is present in many regions of the world. Agriculture consumes around 70% of fresh water at the global level [118]. In areas where the supply of available water limits agriculture production, the issue of deficit irrigation (DI) will gain importance as farmers endeavor to increase the productivity of land. However, due to the limited resources of water, they must cautiously choose crops and irrigation strategies to maximize the crop value and livestock production [119]. According to Yang and Zang [120], it will necessary to improve water use efficiency (WUE) to maintain or even increase crop yield. Hanjra et al. [121] mentioned that the reutilization of wastewater is an issue that needs to be addressed at the global level, but human health and environment protection are insufficient in most of the countries in development. Wastewater reutilization could reduce the water footprint in food production. There is the need to integrate the usage of water reutilization in the governance central framework in order to effectively face the challenges and maximize the potential of this vital resource. The usage of wastewater could be a reliable source for the growth of crops throughout the whole year [122]. It is also necessary to develop new technologies to accelerate the improvement of crops through the enhancement of genotyping and phenotyping and to raise genetic diversity in germplasm [123]. Features that reduce the difference between the potential yield and the real yield in a drought-susceptible environment need to be determined. To achieve this, the main approaches must involve the study of the plant physiology, molecular genetics, and molecular biology [124]. The rapid advance in genomics and proteomics knowledge will indeed be beneficial in refining the methods of transformation and molecular reproduction in order to achieve significant improvement of future crops [125]. In the biostimulants case, the recommendations for future directions of investigation include: finding the most promising substances, isolating active ingredients, and clearly demonstrating the mechanisms that affect the nutrient absorption [126]. The usage of friendly bacteria in combination with humic substances might be useful. For example, its potential has been observed when the plants are subjected to moderate or severe stress. However, there is a lack of studies that have focused on the combined usage of these techniques [127]. Another innovative study has indicated that the application of biostimulants and a low dose of fertilizer (N, P, and K) avoids oxidative stress and improves adaptation to stress conditions without affecting yield [128].

Finally, the high yield management is possible if the agronomic management of soil–plant–microorganism is considered. A challenge for the world’s agronomists involves raising the yield in the same surface while ensuring that the product is safe and of high quality. Adequate management of vegetal nutrition, the usage of biostimulants and strain bio-formulation for the nutrient protection and acquisition must be undertaken in order to achieve this. The use of biostimulants and microorganisms in fertigation programs is necessary since the crops are exposed to temperature changes, drought, frost, pest, and disease exposure. Therefore, offsetting such stress will help to achieve the potential yield in each crop.
